# Functional genomics of HMGN3a and SMARCAL1 in early mammalian embryogenesis

**DOI:** 10.1186/1471-2164-10-183

**Published:** 2009-04-24

**Authors:** Alper Uzun, Nelida Rodriguez-Osorio, Abdullah Kaya, Hongfeng Wang, John J Parrish, Valentin A Ilyin, Erdogan Memili

**Affiliations:** 1 Department of Biology, Northeastern University, Boston, MA, USA; 2 Department of Animal and Dairy Science, Mississippi State University, Mississippi State, MS, USA; 3 Grupo Centauro Universidad de Antioquia, Medellín, Antioquía, Colombia; 4 Department of Animal Sciences, University of Wisconsin-Madison, Madison, WI, USA

## Abstract

**Background:**

Embryonic genome activation (EGA) is a critical event for the preimplantation embryo, which is manifested by changes in chromatin structure, transcriptional machinery, expression of embryonic genes, and degradation of maternal transcripts. The objectives of this study were to determine transcript abundance of HMGN3a and SMARCAL1 in mature bovine oocytes and early bovine embryos, to perform comparative functional genomics analysis of these genes across mammals.

**Results:**

New annotations of both HMGN3a and SMARCAL1 were submitted to the Bovine Genome Annotation Submission Database at BovineGenome.org. Careful analysis of the bovine SMARCAL1 consensus gene set for this protein (GLEAN_20241) showed that the NCBI protein contains sequencing errors, and that the actual bovine protein has a high degree of homology to the human protein. Our results showed that there was a high degree of structural conservation of HMGN3a and SMARCAL1 in the mammalian species studied. HMGN3a transcripts were present at similar levels in bovine matured oocytes and 2–4-cell embryos but at higher levels in 8–16-cell embryos, morulae and blastocysts. On the other hand, transcript levels of SMARCAL1 decreased throughout preimplantation development.

**Conclusion:**

The high levels of structural conservation of these proteins highlight the importance of chromatin remodeling in the regulation of gene expression, particularly during early mammalian embryonic development. The greater similarities of human and bovine HMGN3a and SMARCAL1 proteins may suggest the cow as a valuable model to study chromatin remodeling at the onset of mammalian development. Understanding the roles of chromatin remodeling proteins during embryonic development emphasizes the importance of epigenetics and could shed light on the underlying mechanisms of early mammalian development.

## Background

Early embryonic development is initiated when mature oocytes (MII) are fertilized by spermatozoa. Maternal factors, such as mRNAs, microRNAs and proteins stored in the oocyte, provide the means of support for the first few days of development. The transition from a maternal to a zygotic control of development, called maternal to zygotic transition (MZT), and the activation of the embryonic genome involve chromatin structural modifications that take place during the first few embryonic cell cycles [1]. Embryonic genome activation (EGA) sets the stage for later development [2,3]. Changes in chromatin structure have been characterized throughout the transition from transcriptional incompetence to the minor activation of the zygotic genome at the 1-cell stage and through the major genome activation at the 2-cell stage in murine embryos [4]. In bovine embryos EGA occurs at the 8- to 16-cell stage with extensive programming of gene expression. However, the regulation of chromatin remodeling during EGA still remains a mystery.

Chromatin remodeling is an extensive process occurring during early embryogenesis. An essential property of the embryonic chromatin structure is to prevent the access of the transcriptional machinery to all of the promoters in the genome. The expression of some genes may be mediated by chromatin remodeling proteins. Chromatin remodeling complexes may change the overall pattern of expression of mammalian genes, allowing transcription factors and signaling pathways to produce different genomic transcriptional responses to common signals [5]. This is particularly important for preimplantation embryos starting cell differentiation cascades that will lead to tissue and organogenesis. These changes in chromatin structure generate activation of the transcriptional machinery and gene expression occurring during early embryo development, leading to a unique chromatin structure capable of maintaining totipotency during embryogenesis and differentiation during postimplantation development [3].

The High Mobility Group Nucleosomal (HMGN) protein family is the only group of nuclear proteins that bind to the 147-base pair long nucleosome core particle with no sequence specificity [6]. HMGN proteins are present in the nuclei of all mammalian and most vertebrate cells at approximately 10% of the abundance of histones [7]. They bind as homodimers to the nucleosome and cause chromatin modifications that facilitate and enhance several DNA-dependent activities, such as transcription, replication and DNA repair. This protein family is composed of 3 members, HMGN1 (also known as HMG-14), HMGN2 (also known as HMG-17), and the most recently discovered HMGN3, initially named TRIP7 for its ability to bind the thyroid hormone receptor [8].

In the mouse HMGN1 and HMGN2 have been detected throughout oogenesis and preimplantation development and are progressively down-regulated throughout the entire embryo, except in cell types undergoing active differentiation [9]. Reduction in the levels of HMGN1 and 2 mRNA also occurs during myogenesis in rat, suggesting that down-regulation of HMGN mRNA may be associated with tissue differentiation [10]. Depletion of HMGN1 and HMGN2 in one- or two-cell embryos delays subsequent embryonic divisions. Cells derived from HMGN1-/- mice have an altered transcription profile and are hypersensitive to stress [9]. Experimental manipulations of the intracellular levels of HMGN1 in *X. laevis *embryos cause specific developmental defects at the post-blastula stages. Furthermore, HMGN proteins regulate the expression of specific genes during *X. laevis *development [11]. Several lines of evidence implicate HMGN1 and 2 in transcriptional regulation. Chromatin containing genes that are actively being transcribed has two- to three times more HMGN1 and 2 compared with total chromatin [9].

The human HMGN3 transcript produces two splice variants HMGN3a the long isoform with 99 amino acids, and HMGN3b with 77 amino acids that arises due to a truncation of the fifth exon. Although no HMGN3b protein has been identified in the rat and cow, ESTs with high identity to it suggest that this splice variant may also exist in these species. The cow, mouse, and rat HMGN3a proteins share more than 81% identity with the human HMGN3a protein [8]. The role of HMGN3a has not been studied in mammalian development. Our previous data show that HMGN3a is expressed at similar levels in oocytes and 8-cell bovine embryos [3]. We have detected high HMGN3a mRNA levels in IVF produced bovine blastocysts. Furthermore, HMGN3a was significantly higher in IVF derived blastocysts compared to blastocysts produced by somatic cell chromatin transfer (SCCT), which had lower levels of HMGN3a transcript similar to those detected in somatic cells (unpublished data). Although the exact function of HMGN3a during early embryonic development has not been determined, its role in facilitating chromatin modifications and enhancing transcription, replication, and DNA repair is critical for early embryo development [8].

Another important mechanism in regulation of chromatin structure in the early embryo is mediated by nucleosome repositioning factors, which are ATP-dependent chromatin-remodeling enzymes. Nucleosome repositioning factors use energy released by ATP hydrolysis to alter histone-DNA contacts and reposition nucleosomes to create chromatin environments that are either open or compact. These factors do not involve sequence specific DNA binding sites, but rather are recruited onto promoter regions by specific transcription factors. Nucleosome repositioning factors typically exist as multi subunit protein complexes, like the SWI/SNF (from SWItching and Sucrose Non-Fermenting in yeast) ATP-dependent chromatin remodeling complex [12]. SWI/SNF complexes are thought to regulate transcription of certain genes by altering the chromatin structure around them with their helicase and ATPase activities [13,14]. In mammals, each SWI/SNF complex has any of two distinct ATPases as the catalytic subunit of SMARCA2 (also known as BRM or Brahma) and SMARCA4 (also known as BRG1 Brahma related gene 1) [15]. Both ATPases have important developmental functions. In primates, expression of both subunits remains constant and low throughout embryogenesis until the blastocyst stage [16]. In mouse embryos, Smarca4 transcripts remain at stable levels throughout preimplantation development, while Smarca2 transcripts remain low until the blastocyst stage, when its mRNA levels increase [17]. In porcine embryos, SMARCA2 transcripts are most abundant in germinal vesicle (GV) stage oocytes and decline progressively during embryo development to blastocyst stage [18]. Mutant mice lacking the Smarca4 gene dye at preimplantation while the Smarca2-null mouse mutant is viable and shows a mild overgrowth phenotype [19,20].

Another member of the SWI/SNF family of proteins involved in chromatin remodeling is SMARCA1 (SWI/SNF related, matrix associated, actin dependent regulator of chromatin, subfamily a member 1), considered a global transcription activator and also called SNF2L1. Like other SWI/SNF members, the SMARCA1 protein has a helicase ATP-binding domain. However, since the rest of its motifs diverge from other members of the SWI/SNF family, it has been classified in the ISWI (for Imitation SWItch) subfamily of ATPases, together with SMARCA5. Decreasing levels of SMARCA5 were found during Rhesus monkey embryogenesis from GV oocytes until blastocyst stage. The same study reported low levels of SMARCA1 throughout all stages of embryogenesis except for the 8-cell stage [16].

Members of the SNF2 subfamily of SWI/SNF proteins are characterized by its seven motifs (I, Ia, II, III, IV, V and VI) [21]. SMARCAL1 (SWI/SNF related, matrix associated, actin dependent regulator of chromatin, subfamily a-like 1) is one of the SNF2 members and shows high sequence similarity to the *E. coli *RNA polymerase-binding protein HepA [21]. Recent reports have linked mutations in the SMARCAL1 gene with Schimke immunoosseous dysplasia (SIOD), a human autosomal recessive disorder with the diagnostic features of spondyloepiphyseal dysplasia, renal dysfunction, and T-cell immunodeficiency [22]. The ability of SMARCAL1, to interact primarily with nucleosomes was demonstrated using protein interaction microarrays. SMARCAL1 transcripts are ubiquitously expressed in different human and mouse tissues, suggesting a role in normal cellular functions or housekeeping activities, such as transcriptional regulation [21]. Although no studies have reported the expression of SMARCAL1 during early embryogenesis in mammals, we previously detected a 7-fold increase of the SMARCAL1 mRNA in 8-cell embryos as compared with MII oocytes by using oligonuclotide microarray gene expression analysis and Real Time PCR validation [3].

Additionally, studies on the SWI/SNF complex associated factor SMARCC1 (also called SRG3 and BAF155), a core subunit of the SWI/SNF complex, have highlighted the importance of the ATPase subunits and the whole complex during embryogenesis. In the absence of Smarcc1, mouse embryonic development ceased during peri-implantation stages, indicating that Smarcc1, as well as the chromatin-remodeling process, plays an essential role in early mouse development [23]. SMARCC1 mRNA was found in high levels in GV stage Rhesus monkey oocytes and at very low levels throughout early embryogenesis but was higher later at the hatched blastocyst stage [16].

The limited availability of fully annotated bovine genes has been a limitation for bovine genomic studies. Many bovine proteins are only partially annotated or are based on computational prediction. The objectives of this study were to determine transcript abundance of HMGN3a and SMARCAL1 in mature bovine oocytes and early bovine embryos, to perform comparative functional genomics analysis of these genes across mammals, including humans, annotate and analyze the conserved/non conserved regions of them on the comparative modeled structure.

## Results and discussion

### HMGN3a and SMARCAL1 transcripts are highly expressed in bovine oocytes and early embryos

**Figure 1:**
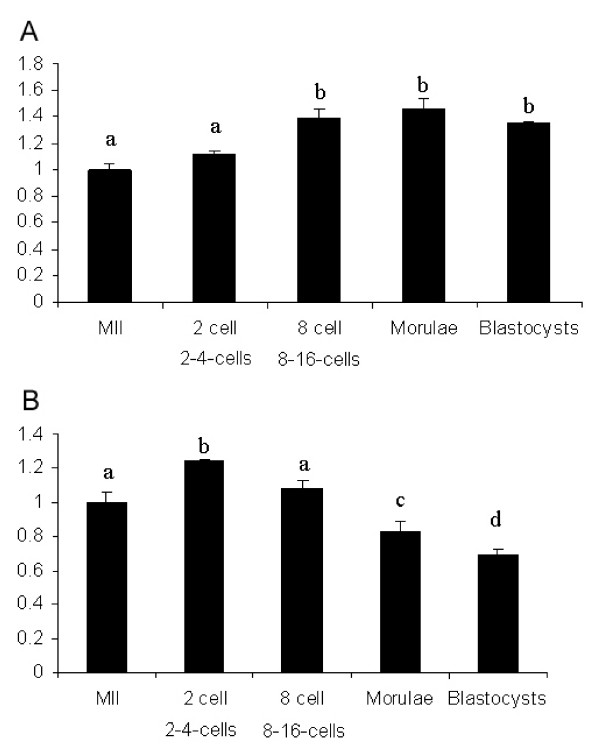
**A. Real Time PCR analysis of HMGN3a**. The mRNA abundance of HMGN3a was determined in bovine matured oocytes, 2–4-cell, 8–16-cell, morula and blastocyst stage embryos. Bars represent relative expression values of 2–4-cell embryos, 8–16-cell embryos, and blastocysts to the expression in MII oocytes. Transcript abundance was normalized to the housekeeping gene, GAPDH. Expression ratio of HMGN3 gene in the 8-16-cell, morulae and blastocysts were significant higher than that during MII and 2–4-cell stages. Different letters represent statistically significant differences (P < 0.05). **B**. **Real Time PCR analysis of SMARCAL1**. mRNA abundance SMARCAL1 was determined in bovine matured oocytes, 2–4-cell, 8–16-cell, morula and blastocyst stage embryos. Bars represent relative expression values of 2–4-cell embryos, 8–16-cell embryos, and blastocysts to the expression in MII oocytes. Transcript abundance was normalized to the housekeeping gene, GAPDH. SMARCAL1 relative expression was similar in MII oocytes and 8-16-cell embryos. However, significantly higher levels of SMARCAL1 in 2–4-cell embryos were observed. SMARCAL1 transcripts decreased significantly in the morula and blastocyst stages. Different letters represent statistically significant differences (P < 0.05).

The mRNA isolated from oocytes and early embryos exhibited typical ribosomal RNA band ratio (28S:18S). Blastocysts showed a ratio of 28S:18S bands closer to 2.0, similar to somatic cells. Using real time PCR, we demonstrated the presence of HMGN3a in bovine MII oocytes, 2–4-cell embryos, 8–16-cell embryos, morulae and blastocysts. HMGN3a transcript abundance was significantly lower in MII oocytes and 2-cell embryos compared to 8-cell, morula and blastocyst stage embryos (Figure 1A). SMARCAL1 transcript abundance was similar in MII oocytes and 8-cell embryos. However we observed significantly higher levels of SMARCAL1 mRNA in 2-cell embryos. SMARCAL1 transcripts decrease significantly in the morula and blastocyst stages (Figure 1B). This result differs from our previous microarray and Real-Time PCR gene expression analysis, which showed that SMARCAL1 was expressed at significantly higher levels in 8-cell embryos compared to bovine oocytes [3]. This difference might be due to the fact that in the current study we have pooled 8- and 16-cell embryos where expression of SMARCAL1 transcripts might be decreasing at the 16-cell stage. The present results confirm the expression of HMGN3a and SMARCAL1 in the bovine oocyte and early embryo. Their confirmed role in chromatin remodeling in other tissues, could suggest involvement of these proteins in the specialized chromatin remodeling process occurring during embryo development.

Smarcc1 (SRG3) expression during mouse oogenesis and preimplantation stages was studied using immunofluorescence and western blot assays. Smarcc1 was present in the nuclei of oocytes during growth and maturation. Following fertilization, Smarcc1 was detected in higher levels in the male pronucleus compared to the female pronucleus. Expression of Smarcc1 was accompanied by expression of Smarca4 and Ini1, other core subunit of the SWI/SNF complex. The expression of these chromatin remodeling factors could suggests a role for remodeling factors in chromatin structure and function during early development [23]. These findings suggest that although the ISWI proteins are widely expressed and play important roles in promoting cellular proliferation and differentiation, they may not play a prominent role during blastocyst formation and may only become key factors during postimplantation life [16].

### Comparative functional genomics analyses of HMGN3a across mammals

**Figure 2:**
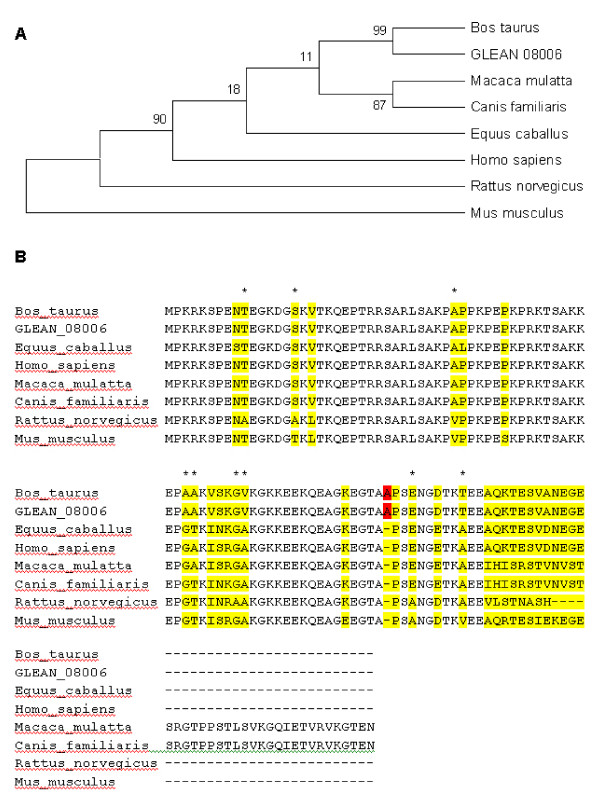
**A. Phylogenetic tree of evolutionary relationships of HMGN3a in 7 mammalian taxa using the Maximum Parsimony method**. The percentage of replicate trees in which the associated taxa clustered together in the bootstrap test (500 replicates) is shown next to the branches. All positions containing gaps and missing data were eliminated from the dataset. **B**. Multiple sequence alignment of HMGN3a. Highlighted regions show substitutions in at least 1 of the 7 species. The alignment includes both the official bovine HMGN3a gene model (GLEAN_08006), in blue, and the bovine NCBI HMGN3a protein (NP_001029676.1). The insertion of alanine in the fifth exon of the bovine protein is marked in red.

Seven mammalian species were used in the construction of a HMGN3a phylogenetic tree (Figure 2A). The percentage of replicate trees in which the associated taxa clustered together in the bootstrap test (500 replicates) is shown next to the branches. All positions containing gaps and missing data were eliminated from the dataset. There were a total of 95 positions in the final dataset, of which 18 were parsimony informative. The most significant observation in multiple sequence alignment of HMGN3a was the insertion of alanine, in the fifth exon of the *Bos taurus *protein, (highlighted in red on Figure 2B). Several substitutions in the bovine sequence were shared by other mammals in the alignment.

HMGN3a constitutes a family of relatively low molecular weight non-histone components of about 100 amino acid residues. *Macaca mulatta *and *Canis familiaris *HMGN3a proteins have longer sequences with regions not shared with the other species. We focused on the regions of the protein shared by all species. Also we showed other alanine substitutions in the alignment (marked with stars) (Figure 2B).

### Comparative functional genomics analyses of SMARCAL1 across mammals

**Figure 3:**
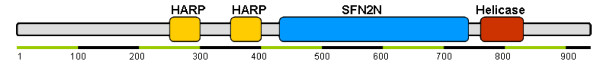
**SMARCAL1 has 4 distinctive domains**. The starting and ending residue numbers are 245–299, 342–396, 437–727, and 741–818.

SMARCAL1 has four conserved domains (Figure 3). The first and the second are two HARP (HepA-related protein) domains of approximately 60 residues long, with single-stranded DNA-dependent ATPase activity. The third conserved domain is a helicase like domain named SNF2 N-terminal domain and the fourth is a helicase C-terminal domain [21].

**Figure 4:**
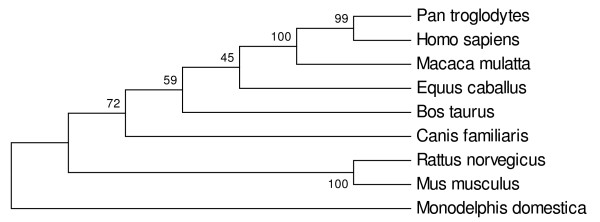
**Phylogenetic tree of evolutionary relationships of the complete SMARCAL1 protein in 9 mammalian taxa, using the Maximum Parsimony method**. The bootstrap consensus tree inferred from 500 replicates is taken to represent the evolutionary history of the taxa analyzed. All positions containing gaps and missing data were eliminated from the dataset. The percentage of replicate trees in which the associated taxa clustered together in the bootstrap test (500 replicates) is shown next to the branches.

**Figure 5:**
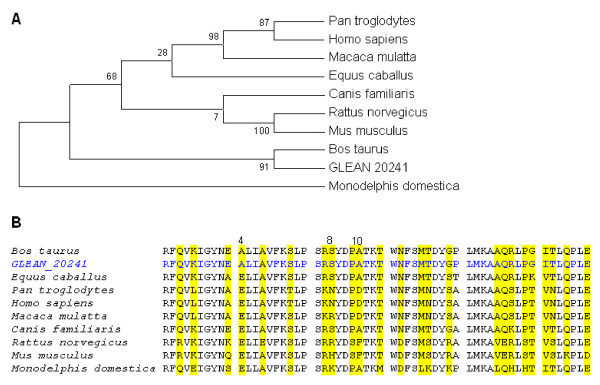
**A. SMARCAL1 first HARP domain phylogenetic tree with the highest parsimony (length = 46)**. The consistency index is 0.95, the retention index is 0.94, and the composite index is 0.90 for all sites and parsimony-informative sites. There were a total of 57 positions in the final dataset, of which 20 were parsimony informative. The percentage of replicate trees in which the associated taxa clustered together in the bootstrap test (500 replicates) is shown next to the branches. **B**. Multiple sequence alignment of the first HARP domain in SMARCAL1. Substitutions in at least one species are highlighted. Numbers on top of the alignment show significant substitutions which are mentioned in the text.

We used the whole SMARCAL1 protein sequences from 9 mammalian species to construct the phylogenetic tree (Figure 4). Branches corresponding to partitions reproduced in less than 50% bootstrap replicates are collapsed. The percentage of replicate trees in which the associated taxa clustered together in the bootstrap analysis (500 replicates) is shown next to the branches. All positions containing gaps and missing data were eliminated from the dataset. There were a total of 856 positions in the final dataset, of which 198 were parsimony informative. We used each one of the four SMARCAL1 conserved domains to build separate multiple sequence alignments and construct separate phylogenetic trees for each domain (Figures 5A, Six part A, Seven part A and Nine part A). Phylogenetic analysis shows that while *Homo sapiens*, *Pan troglodytes*, Macaca *mulatta *are clustering together, Equus *caballus*, Canis *familiaris *and *Bos taurus *have relatively distant position in the tree. *Rattus norvegicus *and *Mus musculus *separated these organisms in the tree. *Monodelphis domestica *becomes the most distant species among 9 mammals in the tree.

**Figure 6:**
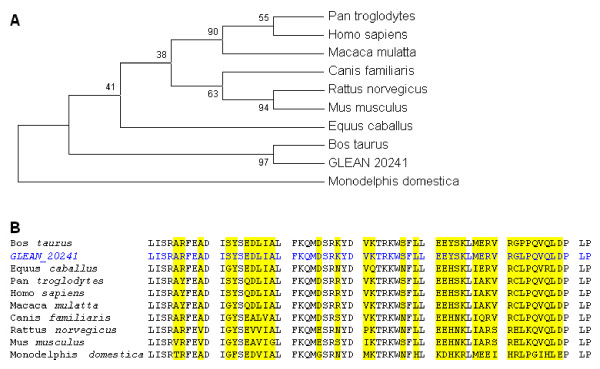
**A. SMARCAL1 second HARP domain phylogenetic tree with the highest parsimony (length = 53)**. The consistency index is 0.84, the retention index is 0.85, and the composite index is 0.77 for all sites. There were a total of 62 positions in the final dataset, of which 17 were parsimony informative. The percentage of replicate trees in which the associated taxa clustered together in the bootstrap test (500 replicates) is shown next to the branches. **B**. Multiple sequence alignment of the second HARP domain in SMARCAL1. Highlighted regions show substitutions occur.

For the first HARP domain, the positions at which substitutions occur are highlighted in yellow (Figure 5B) *Monodelphis domestica *was the most distantly related mammal with respect to this domain. Substitutions were observed in 24 positions. On the 4^th ^substitution, glutamate, a medium size acidic amino acid was substituted by alanine, a small size hydrophobic amino acid in *Bos Taurus*. On the 8^th ^substitution, while *Bos Taurus, Equus caballus*, and *Canis familiaris *have a serine, it is substituted for asparagine in *Pan troglodytes*, and *Homo sapiens*, and for arginine in *Macaca mulatta *and *Rattus norvegicus*. Additionally *Mus musculus *has a histidine, and *Monodelphis domestica *has a lysine at this position. On the 10^th ^substitution, while *Bos Taurus, Equus caballus, Canis familiaris*, and *Monodelphis domestica *have an alanine, a small size hydrophobic amino acid, *Pan troglodytes, Homo sapiens*, and *Macaca mulatta *have aspartate, a medium size acidic amino acid. Both *Rattus norvegicus *and *Mus musculus *have phenilalanine at this position. For the second HARP domain, there were 34 positions with amino acid substitutions in at least one of the species studied. These substitutions are highlighted in the alignment (Figure 6B). Again *Monodelphis domestica *was the most distant species for this domain.

In phylogenetic tree analysis of HARP1 domain, significantly higher bootstrap values were observed for *Rattus norvegicus, Mus musculus, Pan troglodytes *and *Homo sapiens*. In the second HARP domain, high bootstrap values conserved only in *Rattus norvegicus *and *Mus musculus. For both domains Monodelphis domestica observed as the most distant mammalian among 9 species*. When we compared first and second domain of HARP in SMARCAL1 also there was a separation which can easily be identified between the group of *Canis familiaris, Rattus norvegicus, Mus musculus *and the group of *Pan troglodytes, Homo sapiens*, and *Macaca mulatta. Equus caballus *was observed closer to the second group in the first HARP domain.

**Figure 7:**
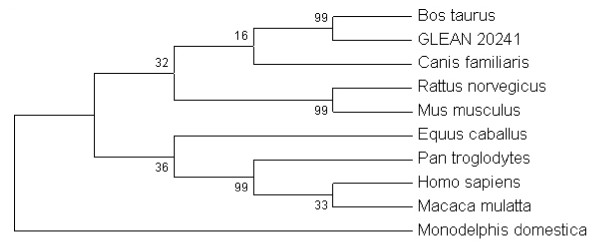
**SMARCAL1 SNF2N domain phylogenetic tree with the highest parsimony (length = 145)**. The consistency index is 0.81, the retention index is 0.79, and the composite index is 0.70 for all sites. There were a total of 290 positions in the final dataset, out of which 39 were parsimony informative. The percentage of replicate trees in which the associated taxa clustered together in the bootstrap test (500 replicates) is shown next to the branches.

**Figure 8:**
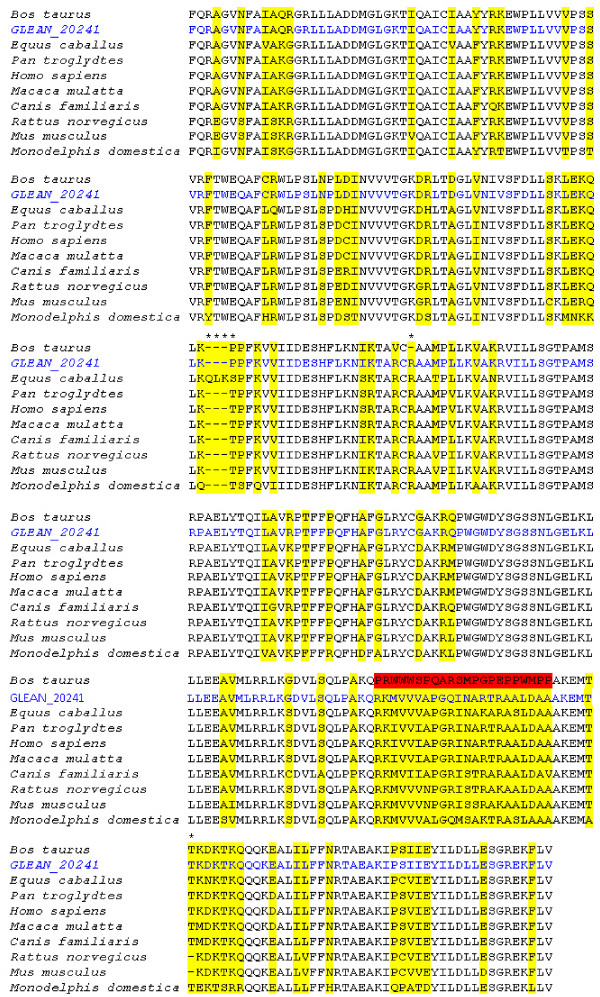
**Multiple sequence alignment of the SNF2N domain in SMARCAL1**. Highlighted regions show where the substitutions occur. The multiple substitutions marked in red in the *Bos taurus *sequence may be due to sequencing errors since the corrected model for this protein (GLEAN_20241, in blue) added to this portion of the alignment only differs from the human sequence in 2 amino acids.

The phylogenetic tree of SNF2N which is the third domain of SMARCAL1 shows similar composition like the first two phylogenetic trees (Figure 7). But the most significant difference is *Canis familiaris *is getting closer to *Bos taurus*. For the last domain of SMARCAL1, one of the clearest observations is lowering of bootstrap values between *Bos taurus *and *GLEAN 20241*, when it is compared to the other phylogenetic trees. Also in the phylogenetic and sequence related analysis 11 parsimony informative sites detected and 46 of the sites are conserved among the species (Figure 8).

**Figure 9:**
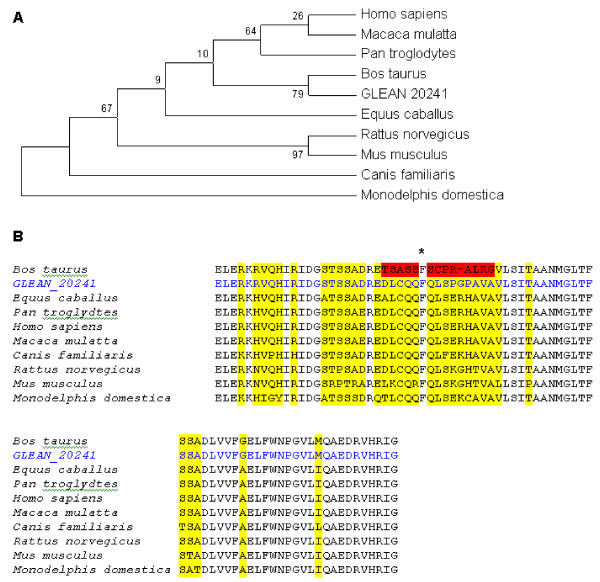
**A. SMARCAL1 helicase C-terminal domain in phylogenetic tree with the highest parsimony (length = 52)**. The consistency index is 0.86, the retention index is 0.81, and the composite index is 0.77 for all sites. There were a total of 78 positions in the final dataset, out of which 11 were parsimony informative. The percentage of replicate trees in which the associated taxa clustered together in the bootstrap test (500 replicates) is shown next to the branches. **B**. Multiple sequence alignment of the helicase C-terminal domain in SMARCAL1. Highlighted regions show where the substitutions occur.

The multiple sequence alignment for the SNF2 family N-terminal domain is shown in Figure 8, with *Monodelphis domestica *as the most distant organism among the other mammals. Positions with insertions and deletions are marked with stars. The first insertion comprises 3 additional amino acids (glutamate, leucine, and lysine) present only in the *Equus caballus*, protein. There is a deletion of the amino acid arginine, present in all species, except for the NCBI bovine sequence. However the GLEAN_20241 does not have the deletion. The amino acid threonine is also absent in both *Rattus norvegicus *and *Mus musculus*. The bovine NCBI sequence showed significant mutations of the third conserved domain marked in red on the alignment. However the sequenced official gene set for this protein (Bovine Genome Database ) shows a higher homology to all species, differing in only 2 amino acids from the horse and human protein. These findings indicate sequencing errors in the currently available bovine SMARCAL1 protein. These errors will likely be corrected with the completion of the bovine genome annotation effort. The bovine helicase C-terminal domain protein shows a deletion (marked with a star) and several substitutions highlighted in red (Figure 9B) that do not exist in GLEAN_20241. These observations point to the need for an update in SMARCAL1 protein sequence currently available at NCBI.

**Figure 10:**
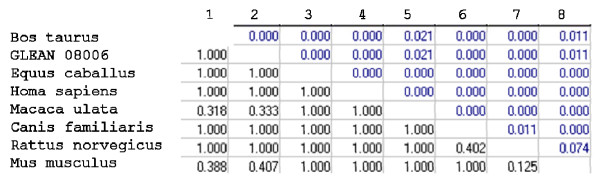
**Probability of rejecting the null hypothesis that HMGN3a sequences have evolved with the same pattern of substitution, as judged from the extent of differences in base composition biases between sequences (Disparity Index test)**. A Monte Carlo test (1000 replicates) was used to estimate the *P*-values, which are shown below the diagonal. *P*-values smaller than 0.05 are considered significant. The estimates of the disparity index per site are shown for each sequence pair above the diagonal. There were a total of 95 positions in the final dataset. None of the P-values were smaller than 0.05. *All positions containing gaps and missing data were eliminated from the dataset*. ***Black colored numbers: ****Probability computed (must be < 0.05 for hypothesis rejection at 5% level)*, ***Blue colored numbers: ****Disparity Index*.

**Figure 11:**
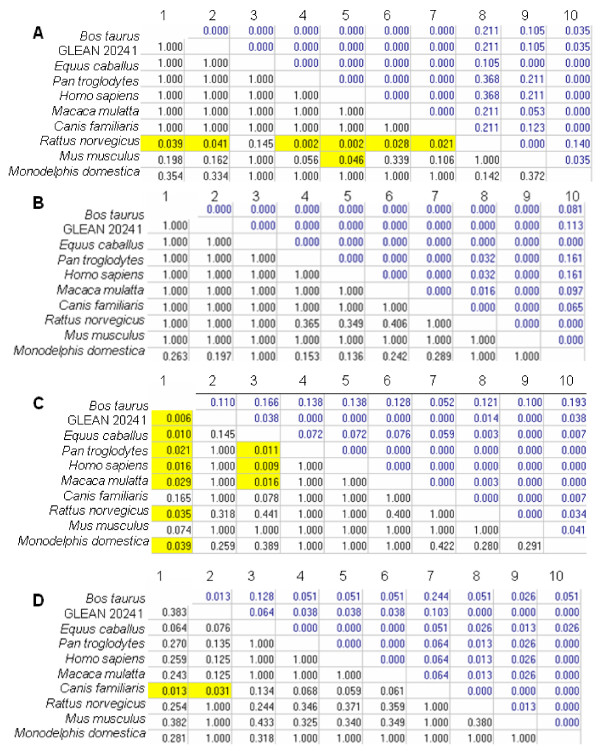
**Probability of rejecting the null hypothesis that the sequences of the SMARCAL1 conserved domains have evolved with the same pattern of substitution, as judged from the extent of differences in base composition biases between sequences (Disparity Index test)**. A Monte Carlo test (1000 replicates) was used to estimate the *P*-values, which are shown below the diagonal. *P*-values smaller than 0.05 are considered significant. The estimates of the disparity index per site are shown for each sequence pair above the diagonal. All positions containing gaps and missing data were eliminated from the dataset. **A**. First HARP domain: there were a total of 57 positions in the final dataset. **B**. Second HARP domain: there were a total of 62 positions in the final dataset. **C**. SNF2N domain: there were a total of 290 positions in the final dataset. **D**. Helicase C-terminal domain: there were a total of 78 positions in the final dataset. ***Black colored numbers: ****Probability computed (must be < 0.05 for hypothesis rejection at 5% level [yellow background])*, ***Blue colored numbers: ****Disparity Index*.

In addition to our analysis, we applied disparity index, ID [24], which measures the observed difference in evolutionary patterns for a pair of sequences. The disparity index for HMGN3a (Figure 10), did not show any significant pairs of species. The disparity index for each domain of SMARCAL1 is presented in Figure 11. In the first HARP domain (Figure 11A) 6 pairs of species (*Bos taurus-Rattus norvegicus, Pan troglodytes-Rattus norvegicus, Homo sapiens-Rattus norvegicus, Macaca mulatta-Rattus norvegicus, Canis familiaris-Rattus norvegicus, and Mus musculus-Homo sapiens*) were considered significant. The disparity index did not observed differences in evolutionary patterns for the second HARP domain (Figure 11B). There were 9 significant pairs in the SNF2 N-terminal domain disparity index (*Bos Taurus-Equus caballus, Bos Taurus-Pan troglodytes, Bos Taurus-Homo sapiens, Bos taurus -Macaca mulatta, Bos Taurus-Rattus norvegicus, Bos Taurus-Monodelphis domestica, Equus caballus-Pan troglodytes, Equus caballus-Homo sapiens, Equus caballus-Macaca mulatta*) (Figure (Figure 11C). In the disparity index for the helicase C-terminal domain only the pair *Bos taurus-Canis familiaris *was significant (Figure 11D).

### Annotation of HMGN3a and SMARCAL1

The official gene model for HMGN3a (GLEAN_08006) was exactly identical to the NCBI protein (NP_001029676.1) with 6 exons and a total of 100 amino acids. No changes were annotated for this protein.

The official gene model for SMARCAL1 (GLEAN_20241) with 937 amino acids differed from the bovine NCBI protein (NP_788839.1) with 941 amino acids, particularly in 2 of the 16 exons. The official gene model for SMARCAL1 showed a higher level of sequence similarity to the human SMARCAL1 protein (NP_054859.2) compared to the available bovine SMARCAL1 protein at NCBI. These results are presented in Table 1. The differences between GLEAN_20241 and NP_788839.1 were caused by an incorrect translation start and several sequencing errors that included 3 amino acid substitutions in exon three, one amino acid substitution in exon five, a sequencing error of 18 amino acids in exon ten, and a sequencing error of 13 amino acids in exon thirteen.

**Table 1:** Pairwise alignment results comparing both the NCBI bovine SMARCAL1 protein and the official gene model for SMARCAL1 (GLEAN_20241) to the human SMARCAL1 protein.

	Bovine NCBI SMARCAL1 vs. Human SMARCAL1	GLEAN_20241 vs. Human SMARCAL1
Similarity Score	3483	3799
Match	74%	79%
Number of Matches	718	764
Number of Mismatches	208	165
Total Length of Gaps	43	33

The official gene model shows a higher sequence homology to the human protein with more matches, shorter gaps, and fewer mismatches.

Annotation of SMARCAL1 mRNA showed that the first HARP conserved domain in SMARCAL1 is composed of the last part of the first exon, the second exon and the first part of the third exon. The second HARP conserved domain is composed of the end of the third exon, the fourth exon and the first part of the fifth exon. The third conserved domain, a helicase like domain named SNF2 family N-terminal domain is composed of the end of the fifth exon and exons 6–12. The fourth conserved domain, the Helicase C-terminal domain is composed of the last part of the twelfth exon and exons 13 and 14. The NCBI protein sequence for the Helicase C-terminal domain may also contain several sequencing errors.

### Analysis of the conserved/non-conserved regions on the comparative modeled structure of SMARCAL1

**Figure 12:**
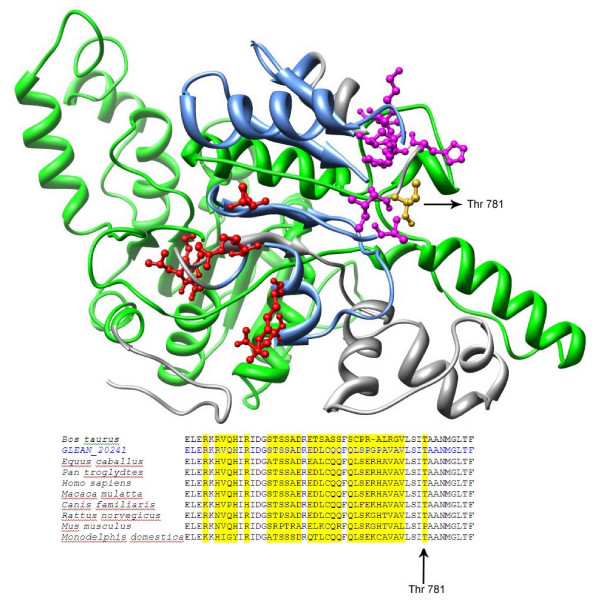
**Based on the domains in the multiple sequence alignment, residues are colored in green and blue**. Green color represents the helicase like domain and blue color represents helicase domain. ATP binding residues are shown in red color and represented as balls and sticks. Nucleotide binding residues are colored in magenta color. The arrows shows the residue which existed in the non-conservative region on the structure and in the MSA, which is indicated in yellow.

Since the protein structures for SMARCAL1 are available for helicase like and helicase domain, a comparative homology model was built on covering only these domains. The percentage similarity between template and protein sequence was 24%. Depending on the multiple sequence alignments, all non-conserved residues were mapped on the modeled structure (Figure 12). SNF2N and helicase C domains have nucleotide binding and ATP binding residues. There are 9 residues responsible for nucleotide binding and 8 residues for ATP binding which were retrieved from the literature [25,26]. These locations were mapped on the modeled structure. In our analysis we showed that all ATP binding residues exist in the conserved regions. Although Threonine781 is among the residues that are responsible in nucleotide binding, falls into the non-conserved region in protein sequence. Multiple alignment results show that in this specific location only one species (in *Mus musculus*) has variation which is Proline (Figure 12). This substitution creates a difference in amino acid side chain polarity as well as hydrophobicity and size at that specific position.

## Conclusion

In our analysis, the bovine HMGN3a and SMARCAL1 showed a higher degree of homology in all studied mammals. This high structural conservation highlights the importance of chromatin remodeling in the regulation of gene expression, particularly during early embryonic development. Understanding the interactions between these proteins and their roles could improve our understanding of epigenetics in reproduction and disease. Appropriate models for the study of chromatin remodeling proteins are essential to understanding this process, particularly in the case of diseases like Schimke immunoosseous dysplasia (SIOD), caused by a mutation in the SMARCAL1 gene. The greater similarities of the HMGN3a and SMARCAL1 proteins in human and bovine could suggest that more attention should be paid to a bovine model in the study of chromatin remodeling.

## Methods

### Production of bovine matured oocytes and early embryos *in vitro*

#### Chemicals and culture media

All chemicals were purchased from Sigma Chemical Company (St. Louis, MO, USA) unless otherwise stated. The synthetic oviduct fluid (SOF, Specialty Media) was used as a base media for embryo culture.

#### *In vitro *maturation (IVM)

Oocytes were collected from 2–8 mm follicles of bovine ovaries obtained from a local slaughterhouse in Wisconsin. Only oocytes containing several layers of cumulus cells and homogenous cytoplasm were selected. Oocytes were washed three times in TL-HEPES and matured in Tissue Culture Medium (TCM) 199 (Gibco/Invitrogen) supplemented with 0.2 mM pyruvate, 0.5 μg/ml follicle-stimulating hormone (FSH; Sioux Biochemicals, Sioux City, IA, USA), 5 μg/ml luteinizing hormone (LH; Sioux Biochemicals), 10% fetal calf serum (FCS, Gibco/Invitrogen), 100 U/ml penicillin and 100 mg/ml streptomycin (Gibco/Invitrogen). Ten oocytes in each 50 μl maturation drop were covered with mineral oil and incubated for 24 h at 39°C in a humidified incubator with 5% CO_2 _[27]. After 24 hours, mature oocytes were washed twice with TL-HEPES. Mature oocytes were randomly selected for either RNA isolation or fertilization. Pools of 100 oocytes were frozen at -80°C on RLT lysis buffer (Qiagen Valencia, CA) until RNA isolation.

#### *In vitro *fertilization (IVF)

Groups of 10 oocytes washed with TL-HEPES were transferred into 44 μl drops of fertilization medium (glucose-free TALP supplemented with 0.2 mM pyruvate, 6 mg/ml fatty acid-free BSA, 100 U/ml penicillin and 100 mg/ml streptomycin). Percoll gradient was used for separation of live spermatozoa in frozen-thawed semen [28]. Briefly, sperm was thawed at 36°C for 1 min, and then carefully layered on top of the Percoll gradient system. Sperm was diluted in TL-HEPES to 5.0 × 10^7 ^cells/ml and 2 μl of diluted sperm were added to the 44 μl fertilization drops, which produced a final sperm concentration of 2.0 × 10^6 ^cell/ml. Fertilization drops (50 μl) were supplemented with 2 μl of 5 μg/ml heparin and 2 μl of PHE solution (20 μM penicillamine, 10 μM hypotaurine, 1 μM epinephrine) and 2 μl of semen (50 × 10^6 ^sperm cells/ml) into the 44 μl fertilization drops [29].

#### *In Vitro *Culture (IVC)

Following 18 hours co-culture of oocytes and sperm, cumulus cells were removed by vortexing the presumptive zygotes in a 1.5 ml Eppendorf tube at high speed for 3 minutes. The cumulus free presumptive zygotes were washed three times in TL-HEPES and approximately 30 cumulus free presumptive zygotes transferred into a 50 μl drops (SOF) under mineral oil for embryo culture. At 72 hpi, 10% FCS was supplemented to each drop except that the 2–4-cell stage embryos were collected earlier than serum addition. In this study, the amount of embryos cleaved was 76.0% while the amount developing to the blastocyst stages was 21.8%.

#### Collection of embryos

Developing embryos of 2–4-cell, 8–16-cell, morulae and blastocysts stages were collected at 44, 100, 120, and 168 hpi, respectively. At the beginning of the embryo culture, the drops were randomly assigned to each developmental stages to collect embryos at the corresponding time. Therefore, the embryos were collected from each drop only for one developmental stage mentioned above. Once the embryos were removed for a specific cell stage, the drops were crossed out to prevent duplicate collection from the same drop. Embryos developing to the corresponding stage were removed from culture drops and washed four times with TL-Hepes with PVP (3 mg/ml). The number of embryos pooled for the development stages of 2–4-cell, 8–16-cell, morulae and blastocysts stages were 50, 50, 20 and 5 per tube, respectively. Total of four tubes were collected from 2 replicates for each development stages. The pooled embryos were frozen at -80°C on RLT lysis buffer (Qiagen Valencia, CA) until RNA isolation.

### Isolation of RNA

Total RNA was isolated from pools of 100 oocytes, 100 2- to 4-cell embryos, 100 8- to 16-cell embryos and 10 expanded blastocysts (evaluated and graded according the International Embryo Transfer Society (IETS) guidelines [30]. Total RNA was isolated using an RNeasy Micro Kit (Qiagen) according to the manufacturer's instructions. Quality of total RNA was estimated using the Bioanalyzer 2100 RNA 6000 picochip kit (Agilent, Palo Alto, CA, USA). RNA quantity and purity were determined using a NanoDrop^® ^ND-1000 Spectrophotometer (NanoDrop Technologies, Wilmington, DE). Total RNA from all groups was normalized to 4 ng and used for cDNA synthesis using SuperScript III Platinum Two Step qRT-PCR kit according to the manufacturer's protocol. Cycling temperatures and times were 25°C for 10 min, 42°C for 50 min, and 85°C for 5 min.

### Real time reverse transcriptase PCR to determine transcript abundance of HMGN3a and SMARCAL1

Complementary DNA was generated using the SuperScript III Platinum^® ^Two-Step qRT-PCR Kit (Invitrogen Life Technologies, Carlsbad, CA) according to the manufacturer's protocol. The kit includes both oligo(dT)20 and random hexamers for cDNA generation. Primers were designed using Primer Premier 5 software (Premier Biosoft International, Palo Alto, CA, USA) spanning 2 exons to avoid genomic DNA amplification (Table 2). Real-time quantitative PCR was performed to assess transcript abundance of HMGN3a and SMARCAL1 relative to the housekeeping gene GAPDH. Quantitative assessment of RNA amplification was detected by SYBR^® ^GreenER™ qPCR SuperMixes for iCycler (Invitrogen Life Technologies, Carlsbad, CA, 11761-100). Five μl cDNA were used for quantitative Real-time PCR reactions according to the iCycler iQ Real-Time PCR instrument (BIO-RAD). Primer concentration was adjusted to 10 μM. The cycling parameters were 50°C for 2 min, 95°C for 8 min 30 sec for denaturation, 40 cycles of 15 sec at 95°C, and 30 sec at 60°C and 30 sec at 72°C for amplification and extension. The melting curve was performed starting at 55°C with 0.5°C increase for 10 sec in 80 cycles. Expression values were calculated using the relative standard curve method. Standard curves were generated using 10-fold serial dilutions for GAPDH and the target gene by measuring the cycle number at which exponential amplification occurred. Results from different groups were analyzed by one-way analysis of variance (ANOVA) by SAS 9.1.3 (SAS Institute inc. Carey, NC). Relative expression software tool (REST) was used to compare all samples of each group. The mathematical model used by REST is based on the PCR efficiencies and the crossing point deviation between samples [31].

**Table 2:** Primers used for Real Time PCR gene expression analysis of HMGN3a, the housekeeping gene GAPDH, and SMARCAL1.

**Gene**	**Primer sequence and position (5' - 3')**	**Product size (bp)**	**Accession Number**
HMGN3a_F	GTTCCAGCCCGTTGCTTTAC (22 – 42)	355	NM_001034504.1
HMGN3a_R	GACCATTCATTCTCCCTCGTTAC (376 – 399)		
GAPDH_F	TGCTGGTGCTGAGTATGTGGT (333 – 354)	295	XM_865742
GAPDH_R	AGTCTTCTGGGTGGCAGTGAT (627–648)		
SMARCAL1_F	CCATCTGCATAGCGGCCTAT (1397–1416)	141	NM_176666
SMARCAL1_R	CGGTTACCACGACGTTGATGT (1537–1517)		

### Comparative functional genomics analyses of HMGN3a and SMARCAL1 across mammals

We retrieved the protein sequences of SMARCAL1 from NCBI by performing protein BLAST [32] against mammalian database using *Bos taurus *SMARCAL1(NP_788839) as our query protein. Sequence data were manipulated with the Friend software [33], a bioinformatics application designed for simultaneous analysis and visualization of multiple structures and sequences of proteins, DNA or RNA. Multiple sequence alignment of 9 mammalian SMARCAL1 protein sequences that are listed in Table 3 were created by using Clustal W [34] under Friend Software. We defined conserved regions based on domains listed in the Pfam [35] database which has conserved amino acid sequence regions. The same steps were applied for constructing HMGN3a phylogenetic tree, for which we used the only available Reference Sequence protein for *Bos Taurus *(NP_001029676). Since the availability of mammalian HMGN3a sequences is limited, we excluded *Monodelphis domestica *from the HMGN3a phylogenetic analyses, which were conducted in MEGA 4 [24]. There are 7 mammalian species used in our analysis of HMGN3a which are shown in Table 4. The Maximum Parsimony method was used for inferring the evolutionary history when creating the phylogenetic trees for both SMARCAL1 and HMGN3a.

**Table 3:** Organisms and protein accession numbers used in multiple sequence alignment of SMARCAL1.

**Organism**	**Protein accession id**
*Bos taurus*	NP_788839
*Homo sapiens*	NP_054859
*Equus caballus*	XP_001490055
*Pan troglodytes*	XP_516076
*Macaca mulatta*	XP_001086469
*Canis familiaris*	XP_536062
*Rattus norvegicus*	NP_001101692
*Mus musculus*	NP_061287
*Monodelphis domestica*	XP_001367889

**Table 4:** Organisms and protein accession numbers used in multiple sequence alignment of HMGN3a.

**Organism**	**Protein accession id**
*Bos taurus*	NP_001029676
*Homo sapiens*	NP_004233
*Equus caballus*	XP_001499146.2
*Macaca mulatta*	XP_001110668
*Canis familiaris*	XP_854051
*Rattus norvegicus*	NP_001007021
*Mus musculus*	NP_080398

### Comparative modeling of SMARCAL1

Since the availability of possible templates for comparative modeling, model was created for only SMARCAL1. PDB-file 1z63 chain A was used as a template for comparative modeling which was sharing 24% with SMARCAL1 protein sequence of *Bos taurus*. The template that was including the residues from 422 to 869 covered two domains of SMARCAL1 and these were helicase like and helicase domains. Comparative modeling was performed by Modeller 9v1 [36]. Structural analysis was done under Friend and model picture was created with Chimera [37].

### Annotation of HMGN3a and SMARCAL1

Annotations were performed using the Apollo software, an interactive tool that enables gene annotators to inspect computationally obtained gene predictions, and edit them by evaluating all the data supporting each annotation [38]. Apollo was successfully used to annotate the *Drosophila melanogaster *genome [39], and was the tool recommended by the Bovine Genome Sequencing Consortium for manual annotation of bovine genes. Apollo software was used to confirm the protein sequence accuracy by transcribing, and translating the DNA, identifying untranslated regions (UTR), translation start, exons, introns, and translation stops. Previous protein information from NCBI or Ensembl, was compared to the GLEAN sequence and errors in the proteins were analyzed in detail. The GLEAN identification number for HMGN3a was GLEAN_08006, and for SMARCAL1 was GLEAN_20241. Annotation of SMARCAL1 and HMGN3a were submitted to the Bovine Genome Annotation Submission Database at BovineGenome.org.

## Authors' contributions

AU performed comparative functional genomics analyses, and drafted the manuscript. NRO designed primers for HMGN3a and SMARCAL1, annotated HMGN3a and SMARCAL1, and drafted the manuscript. HW performed RNA isolations, Real Time RT-PCR experiments and analysis of the results. AK performed in vitro fertilization and culture of bovine oocytes and embryos, and collected the embryos for RNA isolation, and involved with drafting the manuscript. JJP participated in the design of the study, and in the *in vitro *production of the embryos, and reviewed the manuscript. VI participated in the design of the study, in its coordination and helped to draft the manuscript. EM participated in the design of the study, in its coordination and helped to draft the manuscript.
